# Lemierre Syndrome After Influenza A Virus Infection

**DOI:** 10.7759/cureus.115912

**Published:** 2026-09-07

**Authors:** Katherine G Aliotta, Dallin Stevens, Jacqueline M Dovgalyuk

**Affiliations:** 1 Family Medicine, University of Tennessee Health Science Center, Murfreesboro, USA; 2 Saint Louise Clinic, Ascension St. Thomas Rutherford, Murfreesboro, USA; 3 Emergency Medicine, University of Tennessee Health Science Center, Murfreesboro, USA; 4 Emergency Department, Ascension St. Thomas Rutherford, Murfreesboro, USA

**Keywords:** group c streptococcus, influenza a, lemierre syndrome, peptoniphilus asaccharolyticus, septic thrombophlebitis, thrombosis of the internal jugular vein

## Abstract

Lemierre syndrome is a rare condition characterized by septic thrombophlebitis of the internal jugular vein with metastatic septic emboli, most commonly affecting the lungs. We report a case of Lemierre syndrome temporally associated with influenza A infection in a previously healthy 21-year-old male. The patient initially presented to the emergency department with severe headache and tested positive for influenza A. Over the following seven days, his symptoms progressed to neck pain with decreased range of motion, hemoptysis, cough, and persistent myalgias. Despite four additional emergency department visits, his evolving neck symptoms were attributed to influenza-related myalgias and cervical strain. On the day of admission, examination demonstrated neck swelling, tenderness, restricted jaw opening, and pink foamy sputum. Laboratory studies were significant for severe thrombocytopenia, lactic acidosis, and sepsis. Imaging revealed right mastoid effusion, paranasal sinusitis, multifocal pneumonia, and a near-occlusive thrombus of the right internal jugular vein. Blood cultures grew beta-hemolytic group C *Streptococcus* and *Peptoniphilus asaccharolyticus*. The patient was treated with vancomycin, ceftriaxone, clindamycin, and metronidazole. Anticoagulation was initially deferred because of profound thrombocytopenia. Additional therapies included nebulized tranexamic acid, platelet transfusion, corticosteroids, and intravenous immunoglobulin for suspected immune-mediated thrombocytopenia. His hospital course was complicated by metabolic encephalopathy requiring intubation. The patient improved with treatment and was discharged after 17 days on oral antibiotics and rivaroxaban. He was subsequently readmitted 20 days later with a right empyema requiring bronchoscopy, chemical pleurodesis, and decortication. He has remained free of further hospitalization for one year. This case highlights the diagnostic challenges of Lemierre syndrome and the potential for diagnostic anchoring following an initially confirmed viral diagnosis. Although a causal relationship between influenza A infection and Lemierre syndrome cannot be established, clinicians should reassess the diagnosis when patients with confirmed influenza develop persistent or progressive focal symptoms, particularly neck pain or swelling, restricted jaw opening, respiratory symptoms, or systemic deterioration. Early recognition of Lemierre syndrome and prompt treatment are critical to reducing severe complications and morbidity.

## Introduction

Lemierre syndrome is a rare but potentially life-threatening complication of head and neck infection characterized by septic thrombophlebitis of the internal jugular vein with subsequent metastatic septic emboli, most commonly to the lungs [1]. It classically occurs following an oropharyngeal infection and is often described by the clinical triad of pharyngitis or tonsillitis, internal jugular vein thrombosis, and septic embolization [1,2]. Fusobacterium necrophorum is the most frequently identified causative organism, although *Streptococcus*, *Staphylococcus*, and other anaerobic or polymicrobial infections have also been reported [1-3].

Lemierre syndrome predominantly affects otherwise healthy adolescents and young adults, with an incidence of approximately 3.6 cases per million [4]. Patients commonly present with fever, sore throat, unilateral neck pain or swelling, and symptoms related to metastatic infection, including cough, chest pain, dyspnea, or other pulmonary manifestations [1,3]. Diagnosis can be challenging because the initial symptoms may resemble an uncomplicated upper respiratory or pharyngeal infection, while the more characteristic findings of internal jugular vein thrombosis and septic emboli may develop later. Diagnosis is generally based on a compatible head and neck infectious presentation with radiologic evidence of internal jugular vein thrombosis and/or evidence of metastatic septic infection; however, published diagnostic criteria vary, and no universally accepted definition exists [5].

Although Lemierre syndrome is classically associated with tonsillar or peritonsillar infection, other head and neck infections, including sinusitis, otitis media, mastoiditis, parotitis, and other contiguous infections, have also been described [1]. This broader spectrum of potential infectious sources is clinically important when evaluating patients with severe systemic illness and neck symptoms following a recent upper respiratory infection.

Influenza A infection may be relevant as a potential precipitating factor because viral respiratory infections can be complicated by secondary bacterial infections [6]. Prior case reports have described Lemierre syndrome following influenza A infection, although the association remains uncommon and a causal relationship has not been established [7,8].

Our case is notable for the development of Lemierre syndrome following influenza A infection in a previously healthy young adult male, with associated right mastoid effusion and paranasal sinusitis, near-occlusive right internal jugular vein thrombosis, multifocal pneumonia, and polymicrobial bacteremia with beta-hemolytic group C *Streptococcus* and *Peptoniphilus asaccharolyticus*. The combination of influenza A infection, a non-pharyngeal head and neck infectious source, and these bacterial pathogens represents an unusual presentation of this rare syndrome.

## Case presentation

The patient in our case was a previously healthy 21-year-old male who presented to the emergency department five times over seven days. The patient’s clinical course from his initial emergency department presentation through the diagnosis of Lemierre syndrome, subsequent hospitalization, and post-discharge complications is summarized in Figure 1.

**Figure 1:**
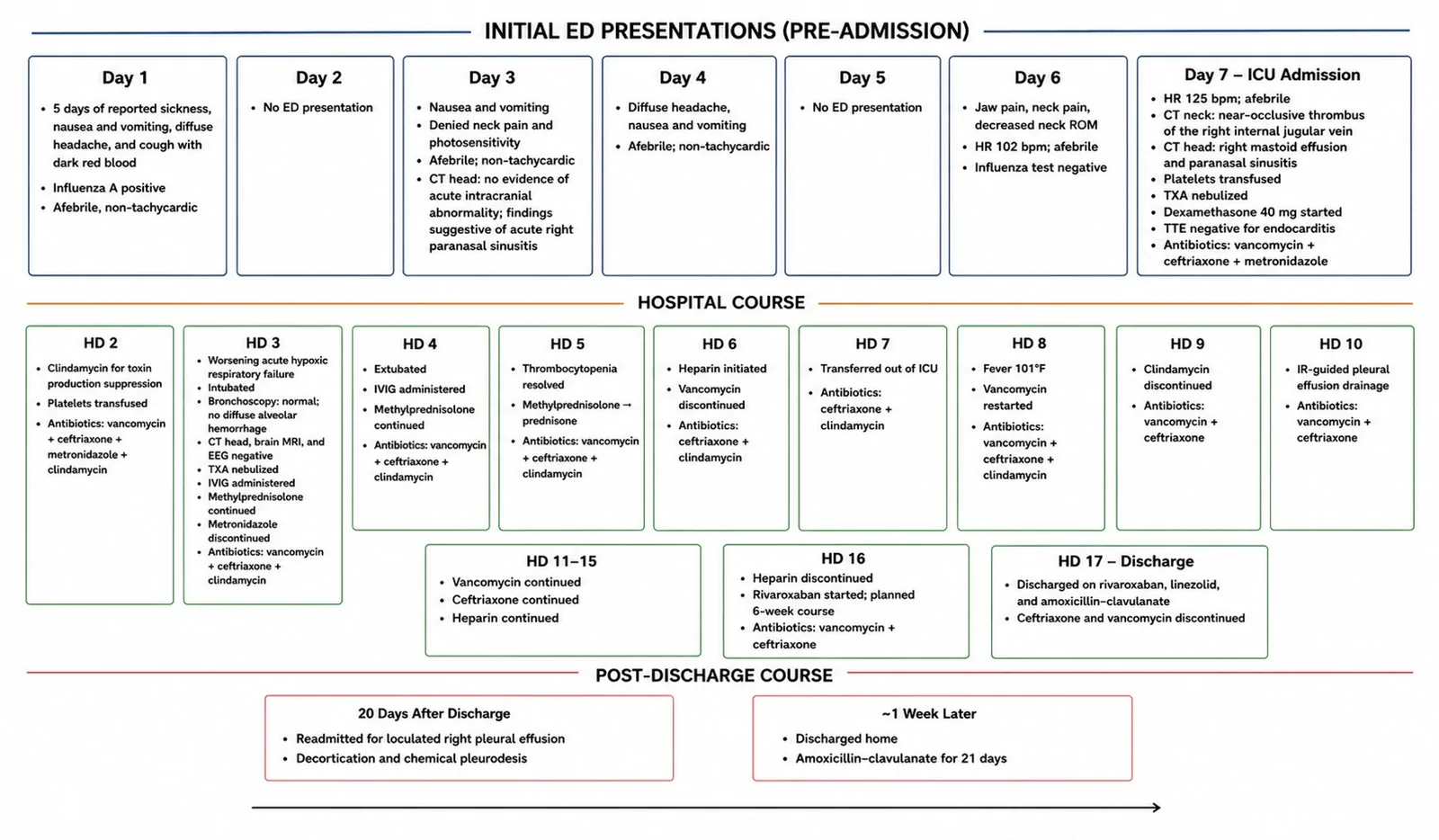
Clinical timeline from initial emergency department presentations through hospitalization and post-discharge course. The timeline demonstrates the evolution from influenza A infection and recurrent emergency department presentations to development of neck and jaw symptoms, identification of right internal jugular vein thrombosis, intensive care unit admission, treatment, and subsequent pleural complications requiring readmission. CT, computed tomography; ED, emergency department; EEG, electroencephalogram; HD, hospital day; ICU, intensive care unit; IJV, internal jugular vein; IR, interventional radiology; IVIG, intravenous immunoglobulin; MRI, magnetic resonance imaging; ROM, range of motion; TTE, transthoracic echocardiogram; TXA, tranexamic acid

On initial presentation to the emergency department, he reported a severe headache, and influenza A was detected by a multiplex nucleic acid amplification assay for influenza A/B, respiratory syncytial virus, and SARS-CoV-2. Over the following days, his symptoms progressed to neck pain with decreased range of motion, cough, hemoptysis, and diffuse body aches that did not improve with ibuprofen or naproxen. Two days after initial presentation, computed tomography (CT) of the head was significant for acute sinusitis of the right paranasal sinuses. Seven days after initial presentation, the patient was admitted to the hospital. At that time, the patient's initial vital signs demonstrated tachycardia with otherwise stable hemodynamics (Table 1). Laboratory evaluation revealed profound thrombocytopenia, elevated inflammatory markers, lactic acidosis, and evidence of a left shift with neutrophilic predominance, consistent with severe sepsis (Table 2). Physical examination was significant for swelling and tenderness to palpation of the anterior and right lateral neck, restricted jaw opening, and pink foamy sputum. The patient was admitted to the intensive care unit for severe thrombocytopenia, multifocal pneumonia, lactic acidosis, and sepsis. Chest radiography demonstrated bilateral interstitial and airspace opacities consistent with multilobar pneumonia (Figure 2). CT of the head revealed right mastoid effusion and paranasal sinusitis, while CT of the neck demonstrated a near-occlusive thrombus of the right internal jugular vein (Figures 3-5). Infectious disease was consulted, which agreed with treatment using vancomycin, ceftriaxone, and metronidazole. Blood cultures were collected prior to antibiotic administration and grew beta-hemolytic group C *Streptococcus *and *Peptoniphilus asaccharolyticus*. Clindamycin was later added on for toxin production suppression. Sputum culture obtained on the day of admission grew upper respiratory flora. Legionella urine antigen was negative. CMV IgM was below the reference range. Varicella Ab IgG was non-reactive. EBV IgM was below the reference range. HIV testing and hepatitis panel were negative. Repeat blood cultures obtained on hospital day 3 were negative.

**Table 1:** Initial vital signs The table presents the initial vital signs on presentation to the emergency department on the day of admission

Vital Sign	Value	Reference Range
Heart rate	129 bpm	60-100 bpm
Oxygen saturation	100%	93-100%
Temperature	98.7°F	97-100.4°F
Blood pressure	142/90	100-180/60-100
Respiratory rate	22	12-20 bpm

**Table 2:** Initial laboratory values The table presents the significant emergency department laboratory results on the day of admission

Laboratory Test	Value	Reference Range
Platelets	4 x 10^3^	130–400 x 10^3^/mm^3^
D-dimer	3.23	0–0.49 mcg/mL
Fibrinogen	685	200–400 mg/dL
White blood cell count	10.7 with left shift and neutrophilic predominance	4.8–10.8 x 10^3^/mm^3^
Lactic acid	3.8	0.5–2.2 mmol/L
Lactate dehydrogenase	337	140–280 U/L
Bilirubin	1.4	0.2–1.2 mg/dL
Alkaline phosphatase	376	40–150 U/L

**Figure 2:**
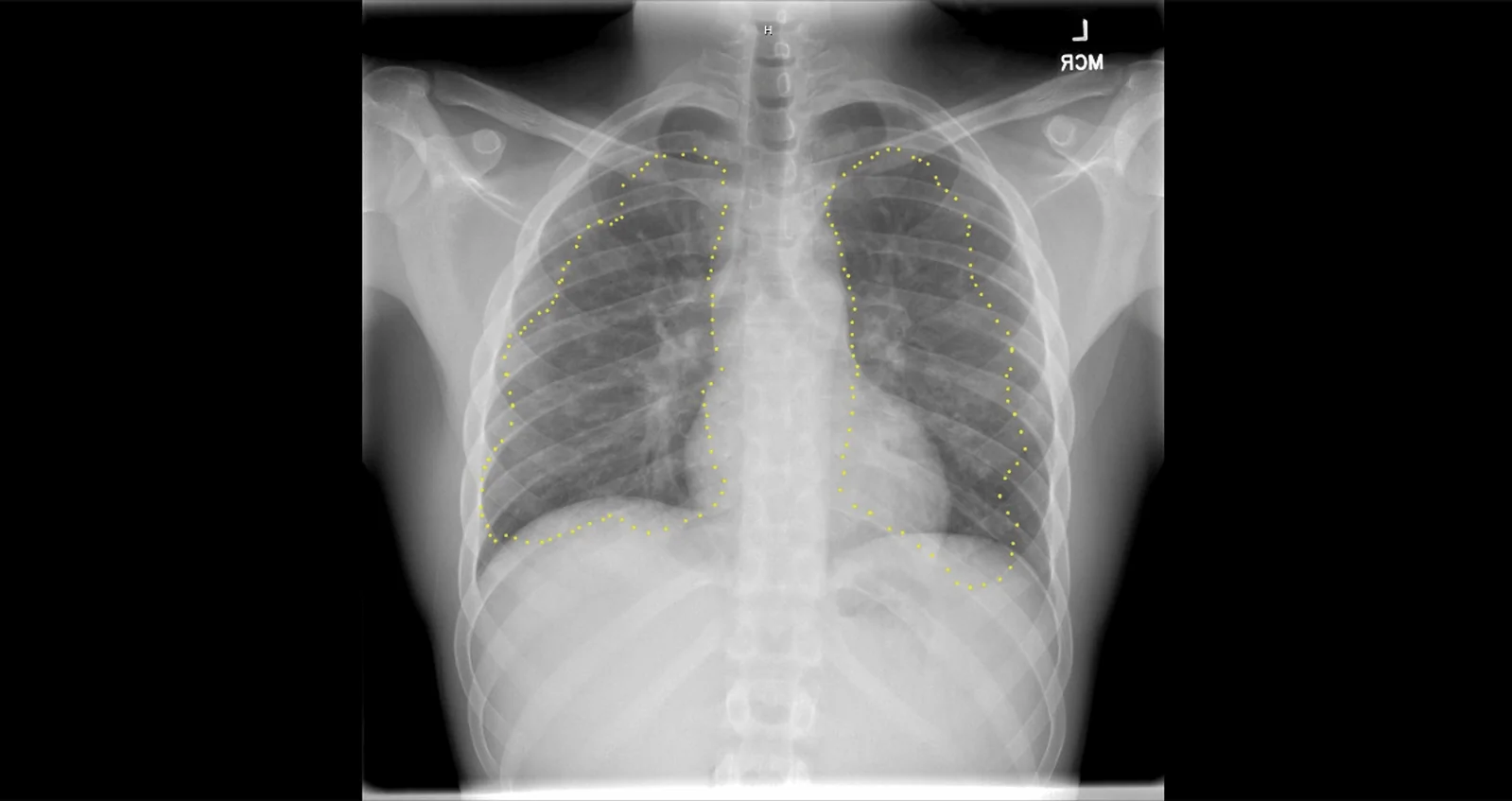
Anteroposterior chest X-ray The image shows bilateral interstitial and airspace opacities, likely multilobar pneumonia.

**Figure 3:**
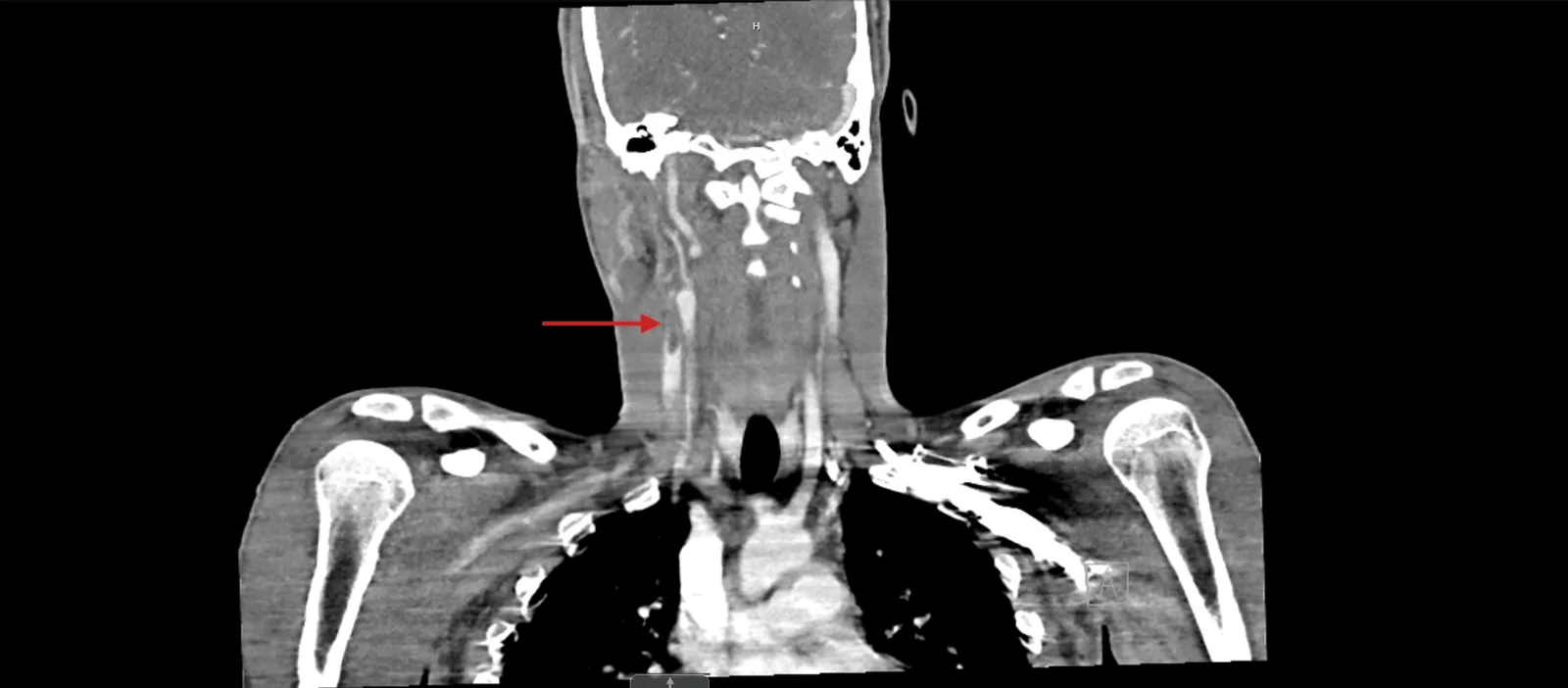
Anteroposterior CT of the neck There is a filling defect present within the right internal jugular vein that is nearly occlusive and extends over a length of approximately 2.6 cm in the neck distal to the clavicle.

**Figure 4:**
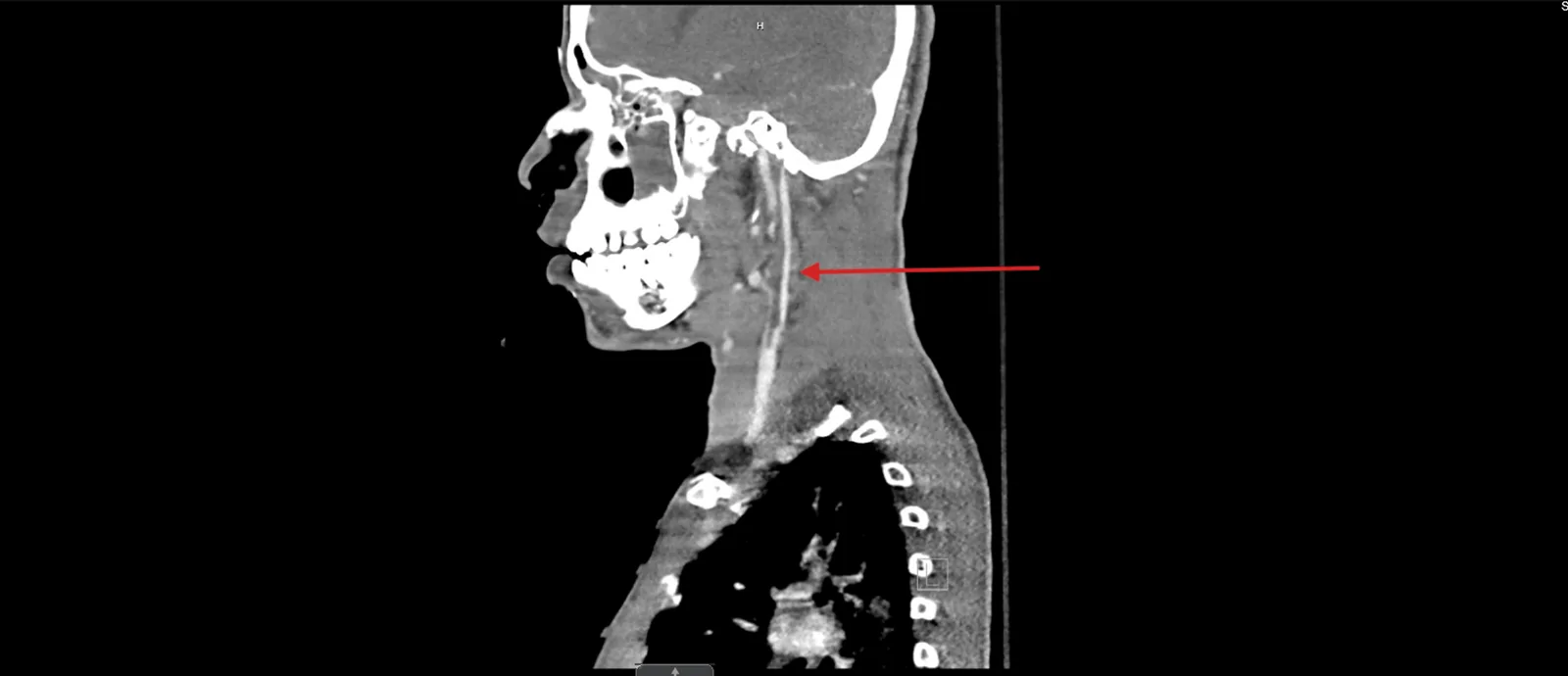
Sagittal CT of the neck There is a filling defect present within the right internal jugular vein that is nearly occlusive and extends over a length of approximately 2.6 cm in the neck distal to the clavicle.

**Figure 5:**
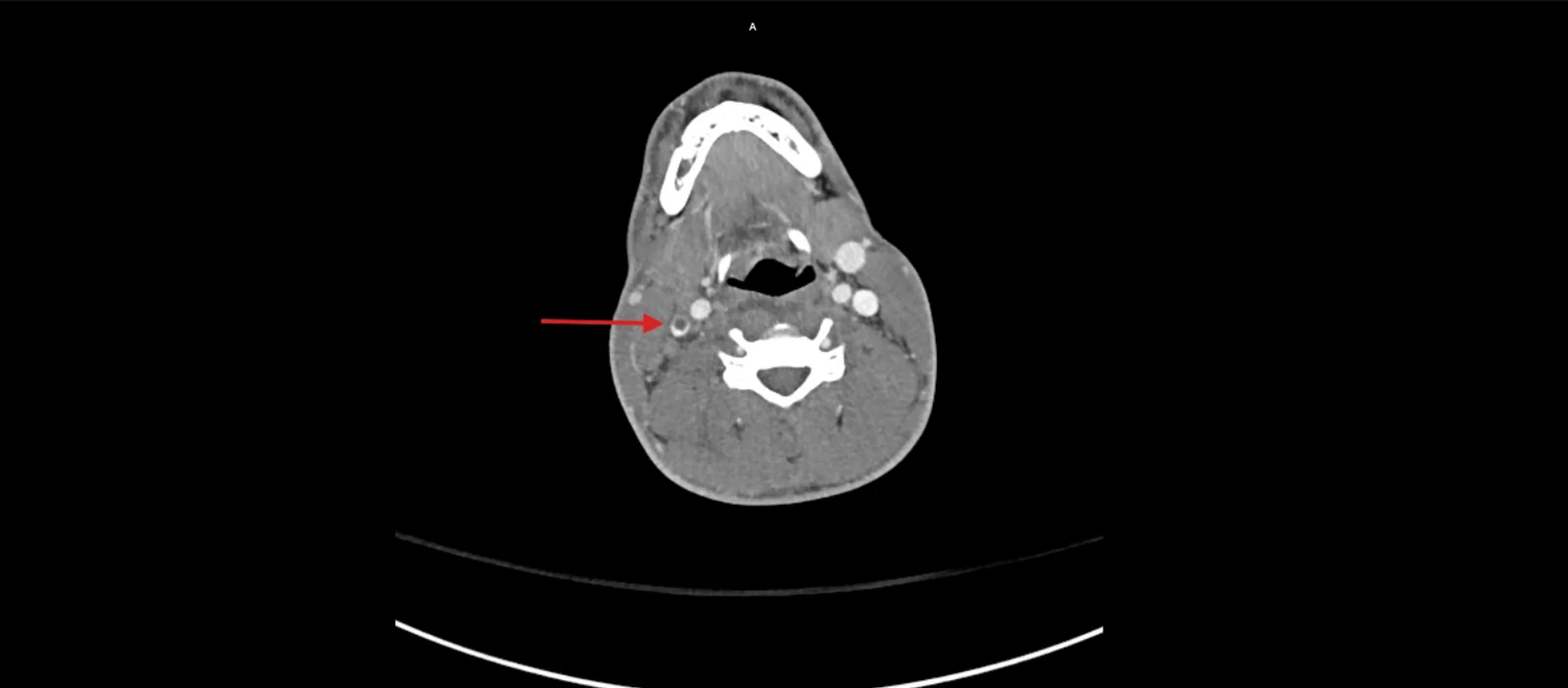
Axial CT of the neck There is a filling defect present within the right internal jugular vein that is nearly occlusive and extends over a length of approximately 2.6 cm in the neck distal to the clavicle.

Anticoagulation was considered for treatment of the septic thrombus but was initially not performed due to severe thrombocytopenia. Hematology was consulted, which suggested that thrombocytopenia was due to overwhelming infection and bacteremia. Nebulized tranexamic acid, seven platelet transfusions, and high-dose steroids were administered over the first three days of the patient's hospitalization due to persistent thrombocytopenia. The patient's platelet count improved to a maximum of 22 x 10^3^/mm^3^ but was not sustained, falling to a nadir of 5 x 10^3^/mm^3^ within hours of platelet transfusion. Intravenous immunoglobulin was administered on hospital days 3 and 4 due to the development of idiopathic thrombocytopenic purpura suspected secondary to overwhelming infection and bacteremia with possible contribution of autoimmune thrombocytopenia. Thrombocytopenia resolved on hospital day 5 with a platelet count of 194 x 10^3^, and heparin was started for anticoagulation on hospital day 6. Anticoagulation was transitioned to rivaroxaban at discharge.

The patient experienced agitation and confusion on hospital day 3 and required emergent intubation as he was unable to maintain adequate oxygen saturation. Work-up for acute metabolic encephalopathy included non-contrast CT head, brain magnetic resonance imaging (MRI) with and without contrast, magnetic resonance venogram (MRV), and electroencephalogram (EEG). Imaging was unremarkable. EEG was suggestive of diffuse cerebral dysfunction of non-specific etiology. Neurology considered his toxic metabolic encephalopathy to be secondary to his severe infection. Lumbar puncture was initially delayed due to severe thrombocytopenia and then deferred given patient improvement with antibiotic treatment. The patient was able to be extubated on hospital day 4.

On hospital day 10, the patient complained of shortness of breath and was found to have a loculated moderate-sized right pleural effusion and underwent thoracentesis. Fluid studies were suggestive of an exudative effusion. Pleural fluid cultures were negative for bacterial growth.

The patient recovered well and was discharged 17 days after admission to the hospital per patient request. The patient was unwilling to stay inpatient for the full recommended course of IV antibiotics despite persistent leukocytosis He was discharged on two weeks of linezolid and amoxicillin-clavulanate. He had completed two weeks of IV antibiotics by this time, and repeat blood cultures were negative. The patient was also discharged on rivaroxaban for six weeks. Unfortunately, he was re-admitted 20 days later due to development of a right empyema seen on CT. The patient underwent bronchoscopy with right chemical pleurodesis and decortication. Pleural fluid cultures showed no growth. The patient was discharged on amoxicillin-clavulanate. Rivaroxaban was discontinued due to the resolution of the septic thrombus on CT and ultrasound imaging. The patient has avoided re-hospitalization for one year.

## Discussion

This case of Lemierre syndrome is notable for its development following influenza A infection and for the diagnostic challenges encountered during the patient's clinical course. The patient's presentation ultimately followed the clinical pattern originally described by Dr. Lemierre: a young adult with a preceding head and neck infection who developed bacteremia, septic thrombophlebitis, and subsequent pulmonary complications including empyema [2]. Importantly, the patient's illness initially appeared consistent with influenza A; however, he returned to the emergency department five times over seven days as his symptoms progressively evolved. This case therefore highlights the importance of reassessing an initial diagnosis when a patient's clinical course does not follow the expected trajectory of an uncomplicated viral infection.

Prior case reports of Lemierre syndrome following influenza infection have been documented [7,8]; however, influenza is not currently considered a well-established predisposing factor for Lemierre syndrome [7]. One case of Lemierre syndrome following influenza A was reported in the Journal of General and Family Medicine in 2019. In that case, a 24-year-old woman developed multiple cavitary lung lesions and left internal jugular vein thrombosis one week after being diagnosed with influenza A and treated with oseltamivir; blood cultures grew *Fusobacterium* [7]. Another case reported in 2011 described a four-year-old child who, following positive influenza A testing and beta-hemolytic streptococcal pharyngitis, developed septic thrombophlebitis of the left jugular vein, although blood cultures remained negative [8].

Influenza may increase susceptibility to secondary bacterial infections through mechanisms including mucosal injury, transient immune dysfunction, and disruption of the normal epithelial barrier. In our patient, influenza A was detected during the initial emergency department presentation, and CT imaging demonstrated acute sinusitis two days later. This temporal relationship between influenza A infection, subsequent sinusitis, and eventual development of Lemierre syndrome raises the possibility that influenza-associated mucosal inflammation or immune dysfunction contributed to secondary bacterial infection in this case. However, a causal relationship cannot be established from a single case. Rather than demonstrating that influenza predisposes patients to Lemierre syndrome, this case adds to the limited number of reported cases describing this temporal association.

A more important clinical lesson from this case is the potential for diagnostic anchoring when a common viral diagnosis provides an initial explanation for nonspecific symptoms. Influenza can reasonably account for symptoms such as headache, myalgias, cough, and malaise; however, our patient's subsequent development of focal neck pain, restricted jaw opening, neck swelling, and hemoptysis represented a significant change from an uncomplicated influenza presentation. His repeated emergency department visits over seven days further demonstrate the importance of reassessing the initial diagnostic impression when symptoms persist, worsen, or evolve in an unexpected manner. In patients with a recent respiratory or head and neck infection, progressive unilateral neck symptoms, trismus, respiratory symptoms, or systemic toxicity should prompt consideration of a deeper bacterial infection and potential septic thrombophlebitis. Early recognition may allow for earlier imaging, antimicrobial therapy, and appropriate multidisciplinary management.

The development of an empyema following treatment for Lemierre syndrome is also consistent with the pulmonary complications associated with this disease. Dr. Lemierre described secondary localization to the lungs as a characteristic manifestation of the syndrome [2]. Pulmonary complications, most commonly related to septic pulmonary emboli, are reported frequently in Lemierre syndrome [1]. In our patient, multifocal pneumonia was identified during the initial hospitalization, followed by development of a right-sided empyema requiring bronchoscopy, chemical pleurodesis, and decortication. This progression illustrates the potential for continued metastatic infection despite initial treatment and emphasizes the importance of close clinical follow-up.

*Fusobacterium necrophorum* is the organism most commonly associated with Lemierre syndrome, although its presence is not required for diagnosis [9,10]. Our patient's blood cultures instead grew beta-hemolytic group C *Streptococcus* and *Peptoniphilus asaccharolyticus*. Streptococcal species have been described as causative organisms in Lemierre syndrome [1,3,9] and were also noted in Dr. Lemierre's original description [2]. Although less common than *Fusobacterium*, these findings demonstrate that Lemierre syndrome should remain a consideration even when blood cultures do not identify the classically associated organism. *Peptoniphilus asaccharolyticus* is an anaerobic organism and, to our knowledge, has not previously been reported as a causative organism of Lemierre syndrome. The polymicrobial nature of the bacteremia in this case further supports maintaining a broad microbiologic differential when evaluating suspected Lemierre syndrome.

At this time, it remains unclear whether influenza A infection directly predisposed this patient to the development of Lemierre syndrome. The temporal association between influenza A infection, subsequent sinusitis, and the development of septic internal jugular vein thrombophlebitis with pulmonary complications is notable, but causality cannot be established. The primary clinical lesson from this case is the importance of diagnostic reassessment when a patient's symptoms persist or evolve beyond the expected course of an initial diagnosis. In patients with recent influenza or other respiratory infections who develop progressive neck pain or swelling, restricted jaw opening, hemoptysis, respiratory symptoms, or systemic deterioration, clinicians should maintain a broad differential diagnosis and consider secondary bacterial infection and Lemierre syndrome. Early recognition of this rare but potentially life-threatening condition may reduce the risk of severe complications from septic thrombophlebitis and metastatic infection.

## Conclusions

This case highlights an uncommon presentation of Lemierre syndrome following influenza A infection in a previously healthy young adult with polymicrobial bacteremia caused by beta-hemolytic Group C *Streptococcus* and *Peptoniphilus asaccharolyticus*. Although *Fusobacterium* species remain the pathogens most classically associated with Lemierre syndrome, this case demonstrates that the syndrome may occur with other bacterial organisms, including mixed aerobic and anaerobic bacteremia. The patient's course, including internal jugular vein thrombosis, multifocal pneumonia, septic pulmonary complications, and subsequent empyema requiring surgical intervention, underscores the potentially severe morbidity and mortality associated with delayed recognition.

Importantly, this case illustrates the potential for diagnostic anchoring following an initially confirmed viral diagnosis. Influenza A was objectively present and provided a reasonable explanation for the patient's initial symptoms. However, his clinical course evolved over repeated emergency department presentations, and the patient developed additional findings, including neck swelling, trismus, hemoptysis, and systemic deterioration. His clinical picture became increasingly inconsistent with uncomplicated influenza. This case emphasizes the importance of reassessing an initial diagnosis when new or progressive symptoms emerge, even when the initial diagnosis is objectively confirmed.

Although a causal relationship between influenza A infection and Lemierre syndrome cannot be established from a single case, the temporal association remains noteworthy. Clinicians should maintain a broad differential diagnosis in patients with recent influenza or other respiratory infections who develop persistent or worsening neck pain or swelling, restricted jaw opening, respiratory symptoms, or signs of systemic infection. Early diagnostic reassessment, appropriate imaging, microbiologic evaluation, and timely antimicrobial therapy are critical to recognizing Lemierre syndrome and reducing the risk of severe metastatic complications and mortality.
